# Experiences of older people with dementia participating in a high-intensity functional exercise program in nursing homes: *"While it's tough*, *it's useful"*

**DOI:** 10.1371/journal.pone.0188225

**Published:** 2017-11-17

**Authors:** Nina Lindelöf, Lillemor Lundin-Olsson, Dawn A. Skelton, Berit Lundman, Erik Rosendahl

**Affiliations:** 1 Department of Community Medicine and Rehabilitation, Physiotherapy, Umeå University, Umeå, Sweden; 2 Department of Community Medicine and Rehabilitation, Geriatric Medicine, Umeå University, Umeå, Sweden; 3 School of Health & Life Sciences, Glasgow Caledonian University, Glasgow, United Kingdom; 4 Department of Nursing, Umeå University, Umeå, Sweden; University of Alabama at Birmingham, UNITED STATES

## Abstract

The objective of the study was to describe the views and experiences of participation in a high-intensity functional exercise (HIFE) program among older people with dementia in nursing homes. The study design was a qualitative interview study with 21 participants (15 women), aged 74–96, and with a Mini-Mental State Examination score of 10–23 at study start. The HIFE-program comprises exercises performed in functional weight-bearing positions and including movements used in everyday tasks. The exercise was individually designed, supervised in small groups in the nursing homes and performed during four months. Interviews were performed directly after exercise sessions and field notes about the sessions were recorded. Qualitative content analysis was used for analyses. The analysis revealed four themes: *Exercise is challenging but achievable*; *Exercise gives pleasure and strength*; *Exercise evokes body memories*; and *Togetherness gives comfort*, *joy*, *and encouragement*. The intense and tailored exercise, adapted to each participant, was perceived as challenging but achievable, and gave pleasure and improvements in mental and bodily strength. Memories of previous physical activities aroused and participants rediscovered bodily capabilities. Importance of individualized and supervised exercise in small groups was emphasized and created feelings of encouragement, safety, and coherence. The findings from the interviews reinforces the positive meaning of intense exercise to older people with moderate to severe dementia in nursing homes. The participants were able to safely adhere to and understand the necessity of the exercise. Providers of exercise should consider the aspects valued by participants, e.g. supervision, individualization, small groups, encouragement, and that exercise involved joy and rediscovery of body competencies.

## Introduction

People living with dementia can have persistence of self [1], ‘a view of a life that is not erased by dementia’ [2], and intact feelings and interpersonal responses [3] even though dementia disorders are chronic, progressive, long-lasting and affect all aspects of life for the person and for their families. Nonetheless, dementia is the leading cause of disability in older people worldwide and the most common reason for people to move to nursing homes [4]. The increasing number of people with dementia is placing a significant burden on health care systems and on informal carers [5, 6]. Consequently, dementia has been considered a public health priority by the World Health Organization (WHO) [7].

Older people with dementia often have impaired balance and walking ability [8], and an increased risk of falls and fractures [9]. Many of them are particularly sedentary [10]. Therefore, they would benefit from exercise and other forms of rehabilitation [11]. Exercise programs for people with dementia have, notably, shown a significant positive impact on walking performance, balance and activities in daily living (ADL) [11–16]. It seems important that the exercise is task specific and progressed to a high intensity, i.e. challenges the individual’s physical capacity [11, 17]. The High-Intensity Functional Exercise Program (HIFE) is one such intervention that has been shown to have beneficial effects for older people living in residential care, including those with dementia, on gait speed, balance, lower limb strength and a slowing of decline in ADLs [14–16, 18–20].

The complexity of dementia symptoms can make it more difficult for older people to participate in exercise programs. Positive outcomes from exercise experiences can increase adherence and participation in exercise, which in turn improves future outcomes [21, 22]. People with dementia can often communicate their views and preferences about what is important to them and it is morally and ethically important to consider those views [23–25]. However, little is known about experiences of participation in high intensity exercise or how positive and negative experiences can act as motivators or barriers to exercise, in older people with dementia in nursing homes. Most previous studies considering experiences of exercise in people in nursing homes have not focused solely on older people with dementia [26–29].

The aim was, therefore, to describe the views and experiences of participation in a high-intensity functional exercise program among older people with dementia in nursing homes.

## Methods

### Context

This qualitative study was undertaken within the context of a randomized controlled trial (RCT) conducted in nursing homes in northern Sweden, the Umeå Dementia and Exercise Study (the UMDEX Study), described in detail elsewhere [16]. The facilities included general and dementia units, all with private rooms and staff on hand, as well as, private apartments with access to on-site nursing and care. Inclusion criteria for participation in the RCT were: dementia diagnosis [30]; aged 65 years or over; dependent on personal assistance in one or more personal ADLs [31]; able to rise from a chair with armrests with assistance of no more than one person; Mini-Mental State Examination (MMSE) [32] score of ten or more; able to hear and understand Swedish language sufficiently well to participate in assessments; and a physician’s approval. All residents who met the inclusion criteria and gave consent to participation were included. Participants were randomized to an intervention based on the HIFE program or to a control activity. The study protocol is published on the ISRCTN registry (ISRCTN31767087).

### Ethics

The UMDEX study, including the present study was approved by the Regional Ethical Review Board, Umeå, Sweden (2011-205-31M).) In accordance with decision from the Ethical Review Board the participants gave oral informed consent to participate, which was affirmed by their next of kin. Before the interviews the participants got renewed verbal information that it was voluntary to participate in the interviews and that they could withdraw at any moment.

### Participants

In the present qualitative study, interviews were performed with 21 participants from 10 exercise groups in the UMDEX Study. The sampling was purposive, i.e. the participants had experience of participation in the exercise program. The participants were selected from a total of 93 exercise participants in the RCT. In addition to the RCT criteria described above the exercise supervisors selected participants who they judged should have the ability to remember and verbally describe their experiences of the exercise session that they had participated in during the last hour. The sampling pursued a variety of experiences, by including men and women from different exercise groups and with a range of dementia diagnoses, ages, functional and cognitive abilities, motivation and intensity levels, and adherence to the intervention (Table 1). All 21 participants identified by the exercise supervisors took part in the interviews.

**Table 1:** Characteristics of the participants interviewed (n = 21).

Age, median (range)	86 (74–97)
Women, n (%)	15 (71)
Barthel Activities of Daily Living Index (0–20), median (range)	13 (5–17)
Mini Mental State Examination, (0–30), median (range)	17 (10–23)
Berg Balance Scale, (0–56), median (range)	37 (4–43)
Mobile indoors:	
Using wheel chair, n (%)	3 (14)
Using walker, n (%)	15 (71)
No walking aid, n (%)	3 (14)
No of drugs, median (range)	9 (0–17)
Alzheimer Disease, n (%)	8 (38)
Vascular, mixed or other dementia, n (%)	13 (62)
Diagnosed depression, n (%)	11 (52)
Previous hip fracture, n (%)	4 (19)
Previous stroke, n (%)	7 (33)
Heart failure, n (%)	3 (14)
Angina, n (%)	6 (29)
Adherence, median % of sessions, (range %)	83 (28–98)
Intensity of strength exercise^a^ (% of attended sessions)	
High / Medium	51 / 40
Intensity of balance exercise^a^ (% of attended sessions)	
High / Medium	80 / 16
Motivation during sessions^a^ (0–4), median (range)	3 (1–4)

^a^ As estimated by exercise supervisors.

### Interviews and field notes

The interviews were carried out individually by the first author (NL) during the last five weeks of the intervention. The interviewer had training in and experience of qualitative research and in conducting interviews. The participants were interviewed directly after an exercise session and in the same room where the session was held in order to facilitate recall of the exercise. They were asked two specific questions, first to describe their experiences of participating in the exercise and later to describe their experiences of exercising in a group. After each question the interview proceeded as a conversation with follow-up questions if needed. The recorded interviews lasted between 12 and 43 minutes (median 20 minutes), and were transcribed verbatim by a person not involved in the study. The interviewer participated in the exercise session preceding each interview and made field notes based on observations in order to get current information, to increase understanding during the process of analysis, and for familiarization between interviewer and participant. Notes were made about, for example, the participant’s exercises, behaviour and comments, and about group dynamics. Field notes also comprised information from exercise diaries of all participants kept by the exercise supervisors.

### Analysis

The analysis was performed using qualitative content analysis, a method for analyzing communication in different steps in a systematic manner [33] and a process of interpretation that focuses on similarities and differences and results in the organisation of the data into categories and themes [34]. The unit of analysis was all interviews. To obtain a sense of the content, the interviews were read through several times by the first author, alongside listening to the interviews to obtain further information from tone of voices and pauses. The next step in the analysis was to identify and divide the interviews into meaning units, a constellation of words or statements that related to the same central meaning. The meaning units were then condensed and labelled with codes. The codes were compared, according to similarities and differences, and codes with similar content were sorted into categories. The seven categories that emerged were further interpreted and abstracted into four themes. Although the interview transcripts formed the primary data set, the field notes were analyzed separately and complemented the interviews. The information from the field notes was considered as to whether it confirmed or not the themes and interpretations. Interpretations of the field note data did not result in new categories or themes. The findings from field notes are presented for each theme with the purpose to further describe and illustrate the themes.

To ensure trustworthiness, some steps of the analysis were also performed by three co-authors (BL, ER, LLO). They worked separately, read through some of the interviews, and then created codes, sorted codes and created categories. At meetings with all five authors the classification and formulation of codes, categories and themes were discussed for all interviews and changes were made until consensus was achieved. The authors each brought a different knowledge base, pre-understanding and experience to the study and data analysis. Both insider and outsider perspectives were represented. The disciplines of the researchers were Physical Therapy (NL, ER, LLO), Nursing (BL) and Exercise Physiology (DAS). Three of the authors (NL, ER and LLO) were involved in planning and testing in the UMDEX study and NL and ER were also supervisors in exercise groups. There were participants from four, of the ten, groups where the interviewer (NL) was one of two supervisors. One author (BL) brought extensive experience in the design and theory of content analysis and the other four authors had extensive experience in falls prevention, exercise interventions, motivation to exercise and working with older people with both physical and cognitive impairments.

### The context of exercise intervention

The HIFE program contains 39 exercises aimed to improve lower limb strength, balance, and mobility [35, 36]. The exercises are performed in functional weight-bearing positions and including movements used in everyday tasks, for example squatting, walking over obstacles, reaching for objects while standing, and climbing stairs. The exercise is intended to be performed at high intensity, i.e. to fully challenge the individual’s ability, and is progressed through increased load or difficulty. High-intensity strength exercises are performed at 8 to 12 repetition maximum (RM), thus progressed by increasing the load when the participant is able to exceed 12 repetitions in an exercise. The load is increased by, for example, stepping higher, rising from a lower chair, or by adding weights onto a belt worn around the waist. High-intensity balance exercises fully challenge postural stability, i.e. are performed at or near limits at which upright position could be maintained, and progressed by, for example, narrowing base of support, altering the surface, or performing more challenging exercises. Physical therapists (PTs) designed an individual program for each participant. Exercises were selected dependent on the degree of functional deficits, and adapted to meet levels of cognition, behavioural and psychological symptoms of dementia, and changes in health and functional status. All participants were individually supervised to encourage high-intensity exercise. Safety was ensured by using a belt, with handles, that participants wore around their waists during balance exercises and thereby the PTs could easily prevent falls. The exercise took place within the facilities, in groups of three to eight participants supervised by two PTs. The sessions lasted approximately 45 minutes, five times per fortnight for four months, with a total of 40 possible sessions per participant. Further insights into the views and experiences of the exercise supervisors delivering HIFE have been previously published [37].

### Characteristics

Baseline assessments of participants were performed by PTs and physicians (Table 1). The Barthel Index was used to assess dependency in ADLs [38]. MMSE to assess cognitive function, and Berg Balance Scale to assess functional balance capacity [39]. Adherence to the exercise sessions was documented as a percentage of the total number of possible sessions (n = 40 per participant). Estimates of achieved training intensity and motivation during the sessions were noted in a structured protocol, by the exercise supervisors (PTs), for each participant after each session. Intensity in strength and balance exercises were estimated as high, medium, or low according to a pre-defined scale [35]. Motivation during the exercise sessions was estimated by the exercise supervisors, on a five graded Likert scale from no motivation to very high motivation, based on interpretations of the expressions, verbal prompts and body language of the participants. The participants’ individual characteristics are shown in Table 2.

**Table 2:** Individual characteristics of the participants.

Interview	Gender	Age	MMSEBaseline/at 4 mo	BBSBaseline/at 4 mo	Adherence^a^Sessions, n	Motivation^b^During sessions,in median
1	Woman	79	16/-	4 / -	33	3
2	Woman	86	20/16	19 / 23	37	4
3	Woman	88	18/17	5 / 19	38	3
4	Woman	96	18/18	43 / 51	31	2
5	Woman	84	17/17	30 / 37	36	3
6	Man	95	14/13	40 / 5	25	4
7	Woman	85	16/14	38 / 44	30	3
8	Man	90	21/16	42 / 40	37	3
9	Man	74	11/6	20 / 44	33	3
10	Woman	79	14/16	37 / 46	13	4
11	Woman	92	23/23	38 / 48	29	3
12	Woman	94	17/10	19 / 31	29	3
13	Man	78	15/19	29 / 30	37	3
14	Woman	86	18/13	39 / 50	32	4
15	Woman	89	19/14	34 / 45	37	3
16	Woman	79	10/8	40 / 42	28	3
17	Man	85	13/15	5 / 3	25	3
18	Woman	97	15/17	21 / 24	26	3
19	Man	84	16/16	41 / 50	36	4
20	Woman	81	18/13	39 / 36	39	4
21	Woman	91	19/18	41 / 47	11	2

MMSE = Mini Mental State Examination (0–30), BBS = Berg Balance Scale (0–54).

^**a**^ Out of 40 possible exercise sessions.

^**b**^As estimated by exercise supervisors on a five graded Likert scale (0–4).

## Results

The analysis of the interviews revealed four themes: *Exercise is challenging but achievable; Exercise gives pleasure and strength; Exercise evokes body memories;* and *Togetherness gives comfort*, *joy*, *and encouragement*. Quotes related to these themes are attributed to participants (described in Table 2) in brackets within the text below. Dots within the quotes indicate pauses. Summaries of field notes from the observations are presented at the end of each theme. In Table 3 examples of quotes and codes, and all categories for one of the themes are shown.

**Table 3:** Examples of quotes and codes, and all categories for one of the themes identified in the interviews.

Examples of quotes	Examples of codes	Categories	Theme
***-****Yes*, *I think it's funny if we can exercise in groups*.* *.* *.* *. *You feel you have a cohesion*.* *.* *. *(12)****-****Well*, *you get contact with some people and it does not hurt*.* *.* *. *it's good*.* *.* *. *you may feel like a stranger else here since this is a completely new area for me anyway*. *(8)*	Provides community Make friends	The exercise provides cohesion	Togetherness gives comfort, joy and encouragement
***-****You feel relaxed*.* *.* *. *and it is very important*.* *.* *. *it is very*, *very important*.. *Very good group*.* *.* *. *very good group*.* *.* *. *(20)*	Well-being in a groupPeaceful group	The group creates comfort	
*-Yes*, *but it’s been great*.* *.* *. *no problem*.* *.* *. *on the contrary*, *fun*. *it has gone smoothly and well and we've laughed and we have been keeping on (14)*	More fun in a group Joy with others	Happiness from meeting others	
*-You get encouraged to do it and you get praised which is fun (21)****-****One gets the inspiration from each other too*. *You see how the others are doing and do as they do (1)*	Spurring in a group Positive comparison Uplifting with others	Encouraging group	
***-****I'm safe when I hear people talk and it is meaningful) (12)* ***-****It is important to be in a group and that someone that take care of you (20)*	Safe in the group Safe with the supervisors	Feeling of security	

### Exercise is challenging but achievable

Participants described that the exercise was challenging in different ways. The necessity of exercise was expressed, alongside the effort required to maintain fitness: *“While it is tough*, *it's useful*…*” (20)*. They stated that they should be physically active and emphasized that they needed to move to keep the body working: “*Probably*, *one should move while living…” (21)*. Some participants mentioned they were getting breathless and that the exercise was strenuous but that exercise was supposed to be that way. To be inactive was described as the worst scenario, causing the loss of both physical function and the mind: *“To sit and lie and just not do anything*… *then you lose your mind*… *I want something to happen and am happy with heavy stuff*, *that’s fine with me*…… *To move that's the alpha and omega …*.. *to just sit and lie*… *that's nothing*. *Yes*, *you lose everything” (9)*.

Other participants commented that they did not like the effort or intensity of the exercises, but still realized it was necessary. One woman said she had been too stubborn and worked too hard and others that the exercise had made them feel insecure and fearful of falling: *“It feels awkward*… *you just have the urge to say NO*, *now we have to put it off completely*… *it is too hard” (13)*. However, some did not think that the exercise was strenuous at all or felt any discomfort and also that the exercise could have been heavier. Participants seemed to adapt to the exercises during the exercise period: *“Well*… *the first time*, *I thought NO*, *this I can never do*… *the second time it went well and the third time it went even better*, *and then it has gone on the fly” (2)*.

Participants, however, mostly voiced that the exercises worked well, particularly as they had been customized for older people and for them individually. They perceived that the exercise supervisors had expertise and helped to ensure that the exercises were adapted to suit them on that particular day: *“It's just that it is directed*, *that is what is so useful*… .. *Yeah but I feel*, *to get instructions to do the movements … that’s what I want*… *that one is cared for*. *It is not done the same way when you are alone” (19)*. There were comments that the sessions were well organized, long enough, and ‘calm’ enough, with many rests and that it could have been risky to exercise by themselves. The participants expressed appreciation that sessions were performed at their facility, so they did not have to travel anywhere. These exercise properties and the supervisors’ expertise seemed to be valued and made the exercise achievable.

The observations during the training sessions showed that although some were skeptical in the beginning of the session and the supervisors sometimes had to encourage them to participate, their eagerness most often increased during the session. As in the interviews, participants sometimes voiced that the exercise was important for their chance to have a break in their everyday routines: *“I think it is important to keep going and that something is happening” (14)*. They also expressed that the exercise was supposed to be strenuous: *“You have to feel it*, *otherwise it is useless” (5)*. There were comments during the exercises reflecting adaptations, such as: *“It is fantastic how you can create different exercises that strain the body” (8)*.

### Exercise gives pleasure and strength

Participants expressed feelings of joy and pleasure about exercising, using words such as ‘funny’, ‘nice’ and ‘agreeable’, and phrases like “*I think it is fun*, *and that’s probably why it goes well*” *(16)*. Some participants said that they always looked forward to the exercise sessions, as they were good for body and soul. They voiced that they were getting re-started, boosted, becoming engaged and coming back to normality, that they were able to do more in their daily lives, and had become more alert and more positive.

It was often described that the exercise dealt with what they needed in *“real life”* and the participants perceived that the exercise improved their fitness: *“Yes it has paid off*… *I think if someone would have told me before that I would stand on tiptoe and knee bend with* *this* *knee*, *I would never have dreamed … but now it just fine” (2)*. One man described it as if exercise was *“saving the power” (9)*. Participants described that the whole body system worked better: *“I am feeling better*… *blood circulation moving differently*… *the heart beats in a different way*… *the blood in the veins*… *you immediately feel that it feels nice*.*” (6)*.

The observations during the sessions showed that the expressions of engagement during the sessions differed between participants. Some seemed determined and focused on their own exercise, while others seemed happy and engaged. Many of the participants said that it felt good, was nice, enjoyable, and that they needed the exercise: *“This is like the health itself”* (19), *“This is so much fun” (8)*. Some expressed that they would like to have more exercise, for example, sessions every day or longer sessions, or did not want to go back to their room when the sessions were finished.

### Exercise evokes body memories

The exercise evoked memories of body movements, physical activities, and heavy jobs that the participants had previously participated in, often many years ago: *“I had brothers*, *and they wanted me to be out jogging with them …” (4)*. Some were also familiar with organized exercise and had been active and competing in sports or had been doing resistance training even into their later years: *“I used to be in a gymnastics group*… *I do not think the difference is so great” (11)*. These previous exercise habits facilitated and motivated for the current exercise: *“Yes*… *because I've been physically active and that is why I … can keep that movement*. *I feel I need to do it because I was so well trained…” (19)*.

The exercise reminded the participants about what they had lost in physical capacity but there were also recognition of movements they actually could do. “*Much has deteriorated … the balance and*… *a lot of my self-confidence might have disappeared because of that…*..*I just had to be able to come back*… *what I have lost I just have to learn again” (5)*.

Some participants expressed happiness that the exercise had reminded their body how to move and they had rediscovered that they were able and could still participate in activities. They could even be proud about what they could perform when exercising: *“To cope with … that you can handle things*… *as an old man it's pretty damn nice to be not completely decayed” (19)*.

The observations during sessions confirmed that several participants had always liked to be physically active and, therefore, they appreciated this exercise. The exercise gave associations to events earlier in life, for example, when a 95 year old man narrated vividly about how his teacher inspired him to be interested in sports: *“He did exactly as you do*. *He made it pleasurable”* (6). During the exercise sessions some participants expressed amazement and pride that they could perform the exercises and that it went so well: *“I think it is fantastic that we can do this!” (2)*. It was also observed that although the participants said that they did not remember the movements, once a movement was initiated they performed the exercises without problems.

### Togetherness gives comfort, joy and encouragement

The participants expressed positive views about being in a group for the exercise and they spoke about this being more fun than exercising alone. It also emerged that they perceived the exercise to be more effective and stimulating in a group than alone *“I do not know if it gives any more*, *but it is perhaps more fun anyway to be with a few people when we do something*…..*It may well spur*… *so you put in more effort”* (5). Loneliness could be frightening and the group offered safety: “*I'm afraid of loneliness” (12)*. Participants expressed that they were pleased with their group, they felt relaxed and at home, and they all got on well together especially if the group was not too large, indeed the size of the groups were considered a good size. It emerged that some were used to group activities and found it natural while others was not used to groups and felt a bit uncomfortable in the beginning: “*I'm used to sitting alone*… *so I get a little surprised when I have to be with people” (3)*.

The importance of connecting with others in the same situation was emphasized. It gave inspiration and the participants were encouraged and their spirits raised by the others in the group: **“***You see and you imitate” (1)*. When others in the group were performing better and had their spirits lifted one person felt crestfallen: *Yes*, *I want to do more than they do*. . . . *one girl is so good so*… *I'm heartbroken that I cannot do as she*… *(18)*.

The participants voiced that the other group members and the exercise supervisors provided encouragement: “*Well it's a boost … I think it is fun to be praised*… *(20)*. It was verbalized that the PTs inspired trust and security in the exercises themselves and in the participants’ ability to do the exercises: *“I know I am safe with the one helping me*… *so I can join in and do the exercise*.*” (12)*

The observations revealed that participants often lit up when they met the others in the group and that the group members interacted with each other. They seemed happy, were joking, having conversations, and showing interest in the others. There were some negative sides of meeting in a group, such as being irritated by those who had a cold or being self-critical when comparing themselves to others: *“Everybody is so good*, *except me” (17)*. Some participants spread a good atmosphere in the group and were very encouraging and confirming toward the others. Praise from the supervisors seemed to lift the participants and they became excited and encouraged and the supervisors were given reciprocal feedback from the participants.

## Discussion

This study describes the views and experiences of older people with dementia in nursing homes who took part in a high-intensity functional exercise program. The depth and richness of the conversations show that older people with dementia, in these settings, are able to describe their experiences of exercise they recently participated in. The interpretations of the interviews revealed four themes of participants’ descriptions of their experiences. The exercise was described as challenging, since it was of high intensity, but also that it was achievable because it was adapted to each participant’s abilities. The importance to themselves of being physically active was emphasized. Further, the findings show that the participants found pleasure in the exercise program and they experienced improvements and feelings of competence. They wanted to exercise to improve physical capacity and expressed a need for improvements, since they had physical impairments. The exercise evoked memories of previous physical activities and the participants rediscovered that they had bodily capabilities. Supervised exercise and the safety that this created was appreciated. Exercising in a group was described as stimulating and spurring and creating coherence with others in the same situation. The study adds important knowledge to already known benefits of the HIFE program among older people with physical and cognitive impairments in nursing homes [14–16, 18–20].

Although there has been increased interest in the views of people with dementia, there is still a lack of studies considering the exercise experiences of older people with moderate to severe dementia living in nursing homes. Among older people living in residential settings where not all had dementia, one study has considered the experiences of taking part in a falls prevention exercise program [40], and another, the experiences of participation in the HIFE program [26]. In one recent study with eight residents with mild to moderate dementia, interviews were performed about their experiences of participation in the HIFE program [27]. All three studies have some findings that are similar to our study, such as perceptions that exercise gave improved physical functions and mood, as well as opportunity of stimulating meetings with other people, and the possibility to be more active [26, 27, 40]. However, in the present study where the majority had moderate dementia, the participants also expressed that intense exercise and effort was necessary to achieve the desired improvements. This is in contrast to a study about physical activity program preferences and perspectives, which suggested that older people with cognitive impairment preferred simple, light and safe exercise when they gave their views about exercise but without participating in particular programs [41].

An important new finding in the present study was that exercising evoked body memories and participants narrated about old days and previous physical activities and capabilities. They were able to reflect about their present situation and expressed the realization that they still had capabilities they thought they had lost and also that they were able to participate and improve. Participants who had previously exercised compared it with the present exercise program and described that they had missed it and also that they needed physical activity. According to the Continuity Theory of Aging, older people strive to maintain continuity in their sense of self and their self-image, despite impaired function, by continuity of physical activity patterns, i.e. remain physically active during their life-span [29, 42]. Other studies have also found that older people with physical and cognitive impairments, as well as people with mild dementia, strive to remain physically active and thereby achieve well-being [26, 27, 43].

Participants voiced their pleasure in meeting others and a sense of coherence of being in a group. They expressed their feelings of being seen, understood and respected by the supervisors and by the others in the group. Older people, with and without cognitive impairment, prefer individually tailored programs undertaken in a group setting [41]. The role of social interaction, such as being included in enjoyable and meaningful activities, and of feeling supported, has previously been described as important for the ability to cope with dementia [44]. Among community-dwelling older people with dementia exercising together with people in the same situation can act as a motivator [45–47] and with a supervisor as a facilitator for exercise [46, 48]. Experiences of the importance of encouragement from the supervisors and the others in the group could be interpreted within the concept of self-efficacy; according to which ‘encouragement from a reliable source’ is important for self-efficacy beliefs [49]. Another aspect important for self-efficacy beliefs is ‘vicarious experience or role models’ [49], which in the present study can be related to participants’ positive recollections of exercising in a group and being stimulated by watching others. A previous study with older adults with mild to moderate dementia, interpreted views according to Bandura´s theory of self-efficacy, and came to similar conclusions about the important role of the exercise group and the supervisors [27]. The role of interaction with others is also, within the theory of selfhood, described as one important facilitator for constructing meaning and define an individuals’ social world. Selfhood is expressed through social interaction [50], and is defined as a state of having a distinct identity and being an individual, distinct from others. Physical activity has been shown to allow people with mild dementia to remain in touch with their former selves, i.e. sustain a sense of continuity in their lives and their selfhood and to feel like healthy and physically capable persons [43]. Kontos (2005) describes a perspective of selfhood, when using the notion of embodied selfhood, ‘which captures the idea that fundamental aspects of selfhood are manifested in the way the body ‘moves and behaves’, and that humans are embodied beings, that the body is a fundamental source of selfhood, and that people with severe cognitive impairment engage with the world through their bodies [51]. In the present study, participants voiced that exercise evoked bodily memories and it seemed like the body had its own memory, since it was observed, in field notes, that participants could perform exercises even though they said that they did not remember the movements. This finding could be reflected to Kontos’ description of the body as a medium of expression, being and knowing, and further that the body has a kind of corporeal awareness that exists below the threshold of cognition [51].

Finally, as reflected in the participants voiced enthusiasm for the exercise supervisors in this study, the importance of supervisor skills, the continuous iterative process of building on existing knowledge, sharing and reflecting, being alert to any alterations needed for individuals that day, communication and building a relationship and trust with residents have been highlighted as important attributes [37]. Instructor qualities have previously been shown to influence attendance to exercise classes [52].

The study has some limitations. The sample was selected, in the sense that all participants had agreed to participate in the RCT study, though, not specifically to the exercise intervention. Further, it was not possible to interview those who had dropped out of the RCT. The transferability to those living with the most severe dementia is limited, since only people with an MMSE score of ≥10 and able to remember and verbally describe their experiences of exercise during the last hour were included in the study. Because of pragmatic reasons all participants were selected before the interviews started, i.e. the participants were not included one by one until saturation was reached. However, saturation within the responses was reached with no new information coming from final interviews. Strengths of the study were, first, that the interviews were performed in the same room where the exercise sessions were held and performed directly after the sessions, which facilitated participant’s recall and for the interviewer to keep them focused. The field observations also facilitated the interview situation, created context, complimented and confirmed the discussions in the interviews, and the participants became familiar with the interviewer. For trustworthiness reasons, the supervisors, who were familiar with the participants, were asked to choose participants for the interviews who they judged had the ability to remember and verbally describe experiences of the exercise sessions. That people with cognitive levels down to MMSE scores of 10 can answer questions about their life and express their current feelings in a valid way is supported in the literature [23–25]. The interviews were performed with a range of individuals, including those who had lower motivation or a low exercise participation rate, to avoid bias. Furthermore, the authors’ different gender, knowledge base, pre-understanding and disciplines was a strength in the process of analysis.

### Conclusions and implications

This study reinforces the positive meaning of exercise to older people with moderate to severe dementia in nursing homes and their expressed pleasure at the opportunity to participate. Participants were able to safely adhere to and understand the necessity of high intensity exercise and progression for improved physical and mental health outcomes. Although they may not always be able to remember an exercise, once initiated they retained a body memory and could perform the exercise with encouragement. Providers of exercise should consider the aspects of delivery that older people with dementia value and appreciate, such as small group sizes, tailoring and adaptation, fun and encouragement, and the rediscovery of body competencies. Future studies should explore motivation in older people with dementia in nursing homes and factors that might affect motivation and attendance to effective exercise provision, such as instructor qualities, staff and facility barriers and family support.
